# Identification of Potential Biomarkers in Glioblastoma through Bioinformatic Analysis and Evaluating Their Prognostic Value

**DOI:** 10.1155/2019/6581576

**Published:** 2019-04-15

**Authors:** Yangmei Zhou, Li Yang, Xiaoxi Zhang, Rui Chen, Xiuqiong Chen, Wenhua Tang, Mengxian Zhang

**Affiliations:** Department of Oncology, Tongji Hospital, Tongji Medical College, Huazhong University of Science and Technology, Wuhan, China

## Abstract

Glioblastoma is a common malignant tumor in the central nervous system with an extremely poor outcome; understanding the mechanisms of glioblastoma at the molecular level is essential for clinical treatment. In the present study, we used bioinformatics analysis to identify potential biomarkers associated with prognosis in glioblastoma and elucidate the underlying mechanisms. The result revealed that 552 common genes were differentially expressed between glioblastoma and normal tissues based on TCGA, GSE4290, and GSE 50161 datasets. Gene Oncology (GO) and Kyoto Encyclopedia of Genes and Genomes (KEGG) pathway enrichment and protein-protein interaction (PPI) network were carried out to gain insight into the actions of differentially expressed genes (DEGs). As a result, 20 genes (CALB1, CDC20, CDCA8, CDK1, CEP55, DLGAP5, KIF20A, KIF4A, NDC80, PBK, RRM2, SYN1, SYP, SYT1, TPX2, TTK, VEGFA, BDNF, GNG3, and TOP2A) were found as hub genes via CytoHubba in Cytoscape and functioned mainly by participating in cell cycle and p53 signaling pathway; among them, RRM2 and CEP55 were considered to have relationship with the prognosis of glioblastoma, especially RRM2. High expression of RRM2 was consistent with shorter overall survival time. In conclusion, our study displayed the bioinformatic analysis methods in screening potential oncogenes in glioblastoma and underlying mechanisms. What is more is that we successfully identified RRM2 as a novel biomarker linked with prognosis, which might be expected to be a promising target for the therapy of glioblastoma.

## 1. Introduction

Glioblastoma (GBM) remains one of the most common aggressive tumors in the central nervous system [1]. Though the incidence of GBM is about 3.19/100,000 population [2], the prognosis is extremely poor because of the complex biological characteristic and limited treatment options. After standard therapy, maximal safe surgical resection is followed by combined radiochemotherapy; still approximately 70% of patients die within two years, while over 90% die within five years [3–6]. Thus, it is critical to comprehend the mechanisms of GBM and develop some more effective therapeutic strategies to improve the outcome of patients.

Over the past years, tremendous studies have been conducted to explore the potential molecular mechanisms, genetics, and pathways responsible for the development and progression of GBM. However, the precise mechanism of GBM remains unclear. Recently, biological information analysis has caused extensive attention and made consistent breakthrough in searching the oncogenes; various biomarkers for diagnosis and prognosis of cancer have been identified [7–9], so it could also be expanded in GBM for better understanding of the underlying molecular mechanism in GBM and finding a clue for new therapeutic targets.

In this study, we performed biological information analysis among 3 profiles (TCGA, GSE4290, and GSE50161), which were downloaded from the TCGA database and the GEO database and identified the differentially expressed genes (DEGs) between the tumor and normal samples. Besides, functional enrichments and the protein-protein interaction (PPI) networks were applied to annotate gene function and screen hub genes. Prognostic value was evaluated by survival analysis. Findings of our study may hint at a potential prognostic biomarker and therapeutic target.

## 2. Materials and Methods

### 2.1. Microarray Data

The mRNA expression profiles and clinical data were downloaded from The Cancer Genome Atlas (TCGA) database (https://cancergenome.nih.gov/), which contains 169 GBM samples and 5 normal samples; the other two profiles, GSE4290 and GSE50161, were downloaded from the Gene Expression Omnibus (GEO) database (http://www.earthobservations.org/index2.php); we extracted 81 GBM samples and 23 normal samples from GSE4290, and 34 GBM samples and 13 normal samples from GSE50161. Both of the GEO data were generated using the GPL570 (HG-U133_PLUS_2) Affymetrix Human Genome U133 Plus 2.0 microarray platform data.

### 2.2. Data Preprocessing and DEGs Screening

We utilized the R statistical software (version 3.4.4; https://www.r-project.org/) and Bioconductor analysis tools (http://www.bioconductor.org/) to process the raw data. The edgeR package in R was used to screen DEGs between tumor and normal tissues with the threshold of false discovery rate (FDR)<0.01, |log_2_-fold  change|  (|log_2_⁡FC|) >2 in TCGA datasets; the corresponding survival information of 159 glioblastoma samples was obtained after deleting the incomplete statistics. For the GEO datasets, we converted the probe-level data into gene expression profiles based on their platform annotation files; the gene expression values were averaged when analogous to multiple probe; DEGs only with the |log_2_⁡FC| >2 and FDR<0.05 were extracted with the limma package after background correction and data normalization. The differences between the two groups were calculated by Student's t test; the* P*-value was adjusted by Benjamini-Hochberg (BH) method.

Venn analysis was utilized to choose the overlap of the DEGs among the three datasets above. As a result, 552 overlapped DEGs in GBM samples were set as a cohort for further exploring.

### 2.3. Function and Pathway Enrichment Analysis

The GO analysis was undertaken from the following three categories: molecular function (MF), biological process (BP), and cellular component (CC). KEGG (Kyoto Encyclopedia of Genes and Genomes) pathway enrichment analysis was also performed to search the key biological pathways the candidate genes were involved in. The functional analysis was conducted through The Database for Annotation, Visualization and Integrated Discovery (DAVID: https://david-d.ncifcrf.gov/) online tools [10] and separately organized by upregulated and downregulated groups. The* p*-value was set at <0.05, which means an enrichment score and represented the significance of the GO or pathways terms.

### 2.4. PPI Network Construction and Hub Genes Identification

The Search Tool for the Retrieval of Interacting Genes [11] (STRING: https://string-db.org/) is an online tool to show the known and predicted interaction relationship between the candidate genes; we used it to develop the overlapped DEGs' s interaction network. Cytoscape software (version 3.6.1, http://www.cytoscape.org/) was applied to visualize the network of these gene interaction pairs with coefficients of |r| > 0.4 and P<0.05, and the hub genes in the network were identified by utilizing the cytoHubba application in it.

### 2.5. Survival Analysis

We performed survival analysis through R language survival package; Kaplan-Meier survival curves were plotted after adjusting relative hazards from the Cox proportion hazards model; P<0.05 was considered to have statistical significance.

### 2.6. Correlation Analysis

The correlation between the expression of RRM2 and other hub genes was evaluated through GEPIA based on TCGA database. Pearson's correlation coefficient analysis was used to define correlations.

## 3. Results

### 3.1. Identification of DEGs

**Figure 1:**
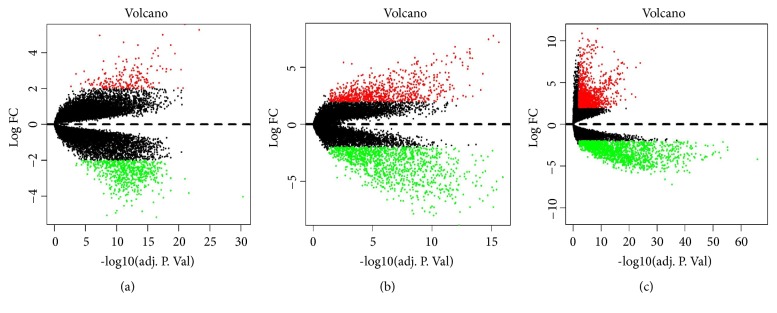
Volcano plot of microarray. Horizontal axis represents log2-fold change, and vertical axis represents adjusted* p* value. (a) GSE4290. (b) GSE50161. (c) TCGA.

**Figure 2:**
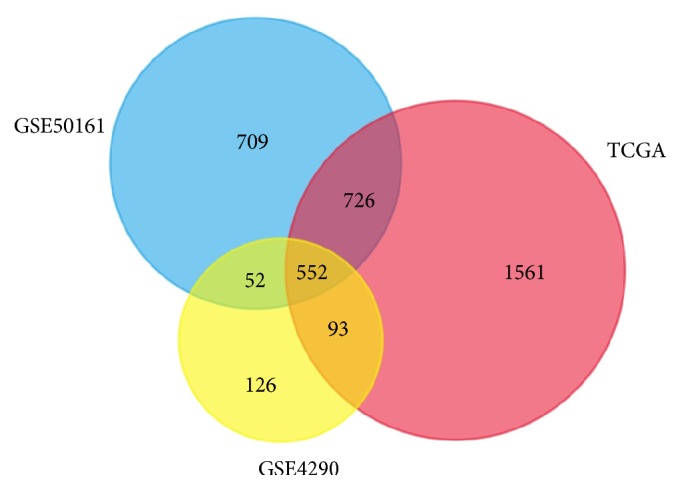
Venn analysis of DEGs. A comparison of 2039, 2932, and 823 DEGs from three datasets revealed 552 common DEGs between GBM and normal tissues.

We filtered a total of 2932 DEGs including 1341 upregulated and 1591 downregulated genes between the tumors and adjacent normal samples from TCGA database, and a total of 2039 DEGs (816 upregulated and 1223 downregulated) and 823 DEGs (212 upregulated and 611 downregulated) from the expression profile datasets GSE50161 and GSE4290 (Figure 1). Finally, 552 common DEGs from three datasets were determined via Venn analysis (Figure 2), including 128 upregulated genes and 424 downregulated genes, which were subsequently used for further investigation.

### 3.2. GO and KEGG Pathway Analysis for Overlapped DEGs

Those common DEGs were well enriched in abundant of functional groups and pathways; the top 5 terms of BP, CC, MF, and KEGG pathway were shown in Tables 1 and 2. In upregulated group, the most enriched terms in each category were GO:0030198 extracellular matrix organization (BP,* P*-value 3.88E-12), GO:0031012~extracellular matrix (CC,* P*-value 6.61E-13), GO:0005201~extracellular matrix structural constituent (MF,* P*-value 2.58E-07), and hsa04512:ECM-receptor interaction (KEGG,* P*-value 2.22E-09), in downregulated group were GO:0007268~synaptic transmission (BP* P*-value 1.62E-31), GO:0045202~synapse (CC* P*-value 1.62E-35), GO:0022836~gated channel activity (MF* P*-value 6.73E-16), and hsa04080:neuroactive ligand-receptor interaction (KEGG* P*-value 3.52E-10).

### 3.3. PPI Network and Hub Genes

**Figure 3:**
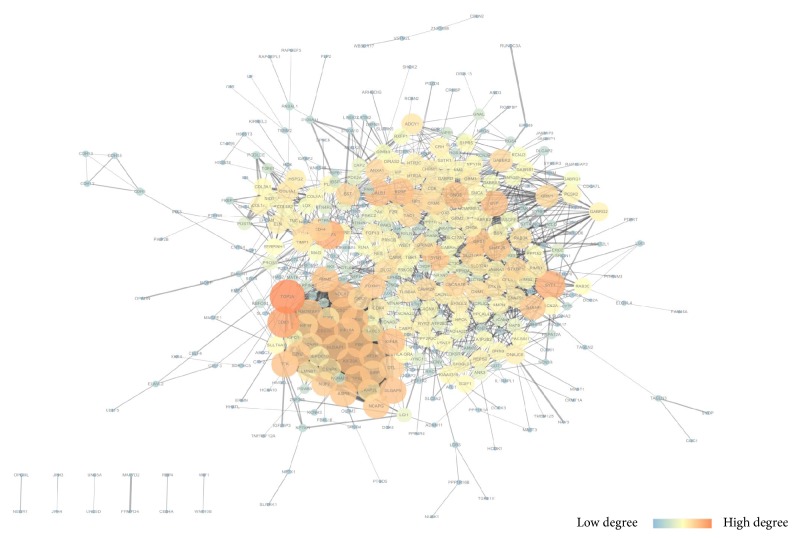
Protein-protein interaction work of overlapped DEGs. Nodes with higher degree are diplayed in larger size and bright orange colour and nodes with lower degree are shown in smaller size and dark blue colour. The edge size is consistent with the coexpression intensity.

**Figure 4:**
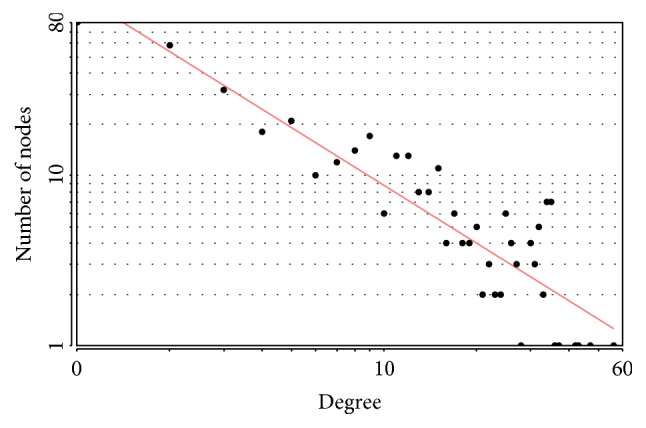
Scatter plot of node degree distribution for overlapped DEGs, R square=0.796, and correlation=0.963.

**Figure 5:**
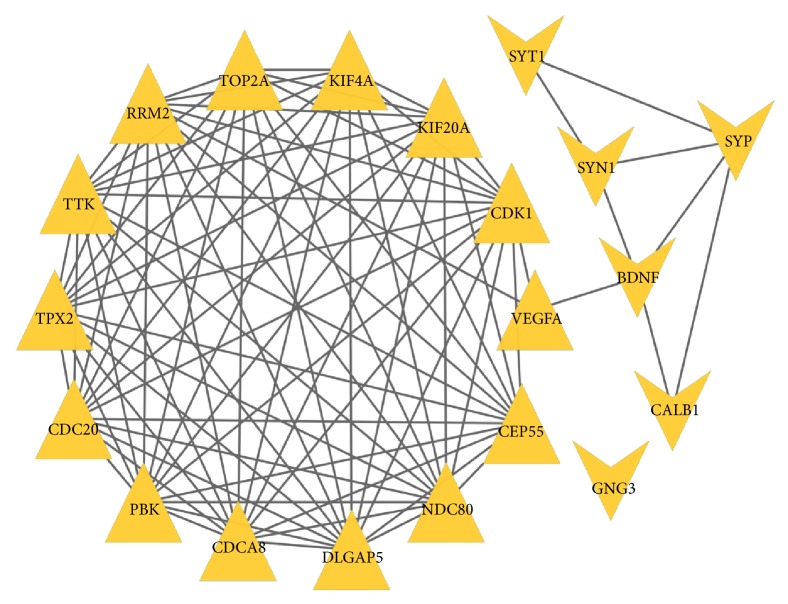
Protein-protein interaction work for 20 hub genes. The shape of triangle represents upregulated genes, the shape of “V” represents downregulated genes.

We mapped the overlapped DEGs to the STRING website, setting P<0.05, coefficients of |r| > 0.4 as a strict threshold, constructed a co-expression network consisting of 401 nodes and 1890 edges (those disconnected genes in the network were deleted); Figure 3 displayed the network visualized by Cytoscape. The distribution of node degrees complied with exponential distribution; R square and correlation coefficient are 0.796 and 0.963 (Figure 4). Then we applied the cytoHubba application to screen out the hub genes in the network; the partial result calculated by multiform algorithm was shown in Table 3. Since the degree is the number of the edges of a gene in the network and represents the interaction pairs with others, it is more likely that the genes locate in a core position and act as a significant function; we adopted the top 20 genes ranked by degree as hub genes; they are CALB1, CDC20, CDCA8, CDK1, CEP55, DLGAP5, KIF20A, KIF4A, NDC80, PBK, RRM2, SYN1, SYP, SYT1, TPX2, TTK, VEGFA, BDNF, GNG3, and TOP2A.  Figure 5 shows the interactions among the 20 hub genes.

### 3.4. Function and Survival Analysis of the Hub Genes

**Figure 6:**
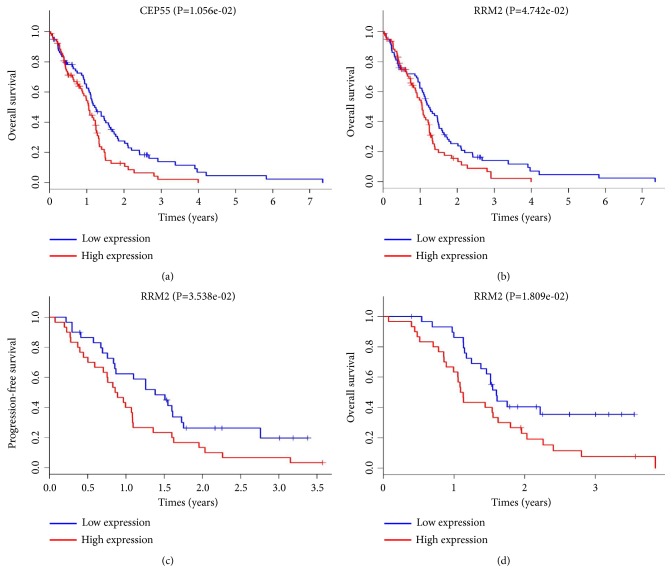
Kaplan-Meier survival curves of GBM patients grouped by median expression level of relevant gene. (a) CEP55 in TCGA. (b) RRM2 in TCGA. (c. d) RRM2 in GSE74187.

By searching the DAVID website, we found the hub genes mainly enriched in the following GO terms: M phase; nuclear division; mitosis; M phase of mitotic cell cycle; organelle fission; microtubule cytoskeleton; mitotic cell cycle; spindle; cell cycle phase; and cell cycle process. CDK1, TTK, CDC20, and RRM2 were found to participate in the cell cycle and p53 signaling pathway (Table 4). Then we utilized the univariate Cox regression analysis and log rank test to draw the survival curves to explore the prognostic related value of the hub genes based on TCGA datasets. Samples with survival information were divided into high expression group and low expression according to the median expression of the candidate genes. CEP55 and RRM2 were discovered to have survival differences (*P*<0.05) (Figures 6(a) and 6(b)); highly expressing CEP55 and RRM2 tends to have poor survival outcomes. To test and verify the result, we did survival analysis on GSE74187, another GEO datasets which contain 60 GBM samples with overall survival (OS) and progress-free survival (FPS) time, with the same calculation strategy; it indicated that the expression level of RRM2 might be related to both OS and PFS (*P*<0.05) (Figures 6(c) and 6(d)), which is consistent with earlier investigation. However, the CEP55 effect cannot be seen in GSE74187, maybe because of the fact that interference comes from limited samples or other confounding factors, so we choose RRM2 for the next step of exploration.

### 3.5. Correlation between RRM2 and Other Hub Genes

Correlation analysis implied that RRM2 expression was positively correlated with DLGAP5 (R=0.79, P<0.05), KIF4A (R=0.78, P<0.05), CDCA8 (R=0.75, P<0.05), TPX2 (R=0.75, P<0.05), KIF20A (R=0.72,P<0.05), PBK (R=0.72,P<0.05), CDC20 (R=0.68, P<0.05), CDK1 (R=0.68, P<0.05), NDC80 (R=0.68, P<0.05), TTK (R=0.68, P<0.05), CEP55 (R=0.62, P<0.05), and TOP2A (R=0.58, P<0.05), while there was insignificant correlation with SYP, VEGFA, BDNF,CALB1, SYT1, GNG3, and SYN1 (supplementary material 1).

### 3.6. Exploration of RRM2

**Figure 7:**
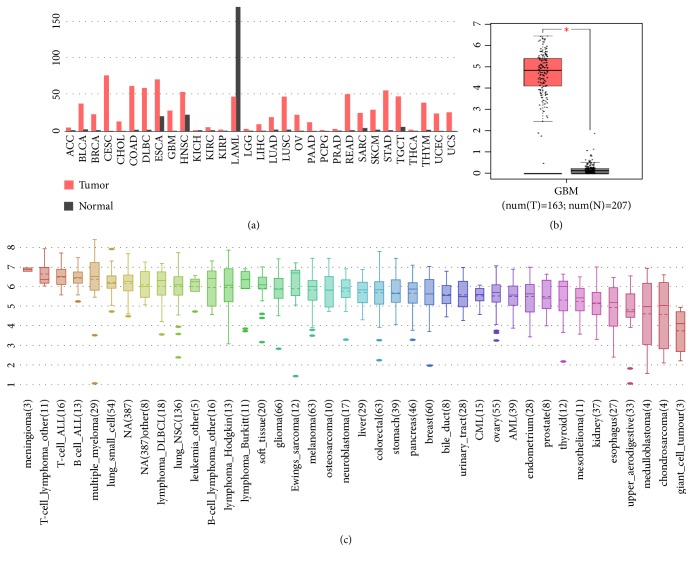
The expression level of RRM2 in different types of cancers and cell lines. (a) In pan-cancer tissues and normal tissues. (b) In GBM tissues and normal tissues. (c) In multitumor cell lines.

**Figure 8:**
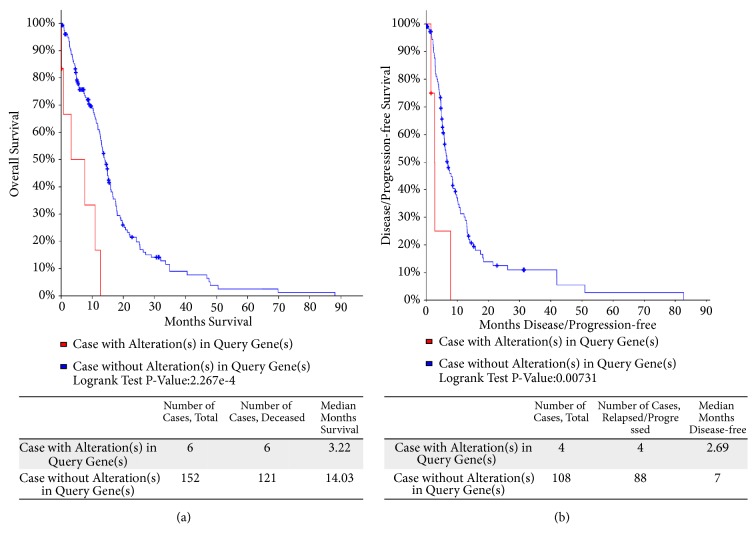
Kaplan-Meier survival curves of GBM patients grouped by RRM2 alteration according to cBioPortal. (a). OS curves. (b). PFS curves.

**Figure 9:**
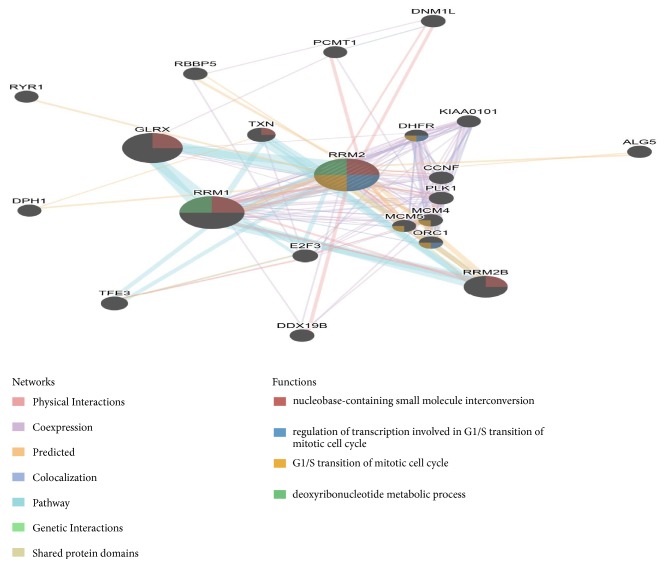
PPI network of RRM2 generated by using GeneMANIA. The network edges refer to the interaction types between gene pairs and the colour filled in the nodes refers to a pathway the gene node participated in.

According to the GEPIA website (http://gepia.cancer-pku.cn/), we observed that RRM2 was differently expressed in many types of tumor tissues including GBM compared to normal tissues (Figure 7(a)), which further indicated it may contribute to the occurrence and development of cancer.  Figure 7(b) shows the different expression level of RRM2 between the GBM samples from TCGA and normal brain samples matched from GTEx datasets (https://gtexportal.org/home/), which contain more abundant normal samples compared to TCGA database. Apparently GBM patients expressed higher level of RRM2. Besides, RRM2 were highly expressed in GBM cancer cell lines (Figure 7(c)) as demonstrated from CCLE analysis (https://portals.broadinstitute.org/ccle/), while the prognostic was worse according to previous research. To explore the probable regulated mechanisms of RRM2 in GBM, we explored the cBioPortal (http://www.cbioportal.org/) and found that RRM2 was altered in 4% of GBM cases from TCGA cohort; the main type of genetic alteration is mRNA upregulation. Correspondingly, those with RRM2 altered seem to have poor outcome (Figure 8). We annotated the biological processes of RRM2 by employing GeneMANIA (http://genemania.org/), a freely available and effective web interface for functional prediction of genes; as is shown in Figure 9, RRM2 interacted with 20 proteins and was mainly involved in the following functions: nucleobase-containing small molecule interconversion; regulation of transcription involved in G1/S transition of mitotic cell cycle; G1/S transition of mitotic cell cycle; and deoxyribonucleotide metabolic process. Considering that, we infer that RRM2 may affect the processes of GBM via regulating mitotic cell cycle and nucleotide metabolism.

## 4. Discussion

Glioblastoma (GBM) is the most malignant glial tumor in brain with devastating prognosis because of its complex biological behaviors and limited strategies for therapy; people with GBM suffer from tumor progression or recurrence. Recently, advances in genomic analysis help us form a comprehensive insight into the mechanisms of GBM and identify potential biomarkers for GBM prognosis and therapy. In the present study, we performed gene expression profile analysis among three datasets: TCGA, GSE4290, and GSE50161, totally included 284 GBM and 41 normal samples. At last, the overlapped 552 DEGs among three datasets were identified, of which 128 were upregulated and 424 were downregulated. GO annotation indicated that the upregulated genes were mainly manifested in cell cycle, mitosis, and extracellular structure organization; the downregulated genes were primarily related to neurological system process, cell-cell signaling, and synaptic transmission. KEGG pathway shows that the upregulated genes were associated with ECM-receptor interaction, focal adhesion, complement and coagulation cascades, cell cycle, and p53 signaling pathway; the downregulated genes were associated with neuroactive ligand-receptor interaction, calcium signaling pathway, long-term potentiation, ErbB signaling pathway, and MAPK signaling pathway. These results indicated that the pathogenesis of tumor is a complex biological process driven by specific genes and epigenetic changes. Abnormal regulation of multiple genes will promote the occurrence and development of GBM through different mechanisms.

To further screen core genes, we constructed a protein-protein interaction network consisting of 401 nodes and 1890 edges through STRING; then we focus our attention on the 20 core genes in the network picked out by cytoHubba according to their degree. GO annotation indicated the 20 hubgenes mainly took part in cell mitosis and effect the cell cycle; among them, CDK1, TTK, and CDC20 were enriched in cell cycle pathway; CDK1 and RRM2 were enriched in p53 signaling pathway. As is known to all, those pathways play a vital role in carcinoma. p53 signaling pathway is composed of a set of genes and their products that response to a variety of intrinsic and extrinsic stress signals in apoptosis, cellular senescence, or cell cycle arrest style [12]; dysregulation of the genes in the p53 network might disrupt the fidelity of DNA replication and cell division and present a greater risk of carcinogenesis [13]. Evidently, rapid proliferation is indispensable for tumor cells to maintain growth and invasion; disorders in the regulation of cell cycle contribute to the initiation and progression of tumor [14]. The hallmarks of rapid proliferation and high aggressiveness of GBM make identifying cell cycle pathway and p53 signaling pathway as a predominant signalling pathway in GBM reasonable. Further unravelling of the precise molecular mechanism about those pathways and relevant genes may be necessary for future efforts.

Present survival analysis revealed that RRM2, one of the hub genes in the coexpression network, was associated with the prognosis of GBM patients; patients with high expression of RRM2 showed shorter overall survival time than those with low expression level. Besides, 4% of TCGA GBM samples detected out RRM2 alteration according to cBioPortal; however, this alteration seems to relate to poor outcome. Hence, we hypothesized that RRM2 could be a potential prognostic factor of patients with GBM. Ribonucleotide reductase (RR), a rate-limiting enzyme which catalyzes the formation of deoxyribonucleotides from ribonucleotides, consists of ribonucleotide reductase subunit M1 (RRM1) and ribonucleotide reductase subunit M2 (RRM2). Over the years, studies continually found that RRM2 was overexpressed in various tumors, such as pancreatic cancer [15], neuroblastoma [16], thyroid malignant tumor [17], breast cancer [18], melanoma [19], lung cancer [20], prostate carcinoma [21], and hepatocellular carcinoma [22]; elevated RRM2 activity played a vital role in tumorigenesis, progression, and invasion and can serve as prognostic markers in some instances. Inhibition of RR can break the balance of ribonucleotide and deoxyribonucleotide level, effecting the DNA synthesis and repair and inducting cell cycle arrest and apoptosis [23–25], making RR an attracting cancer therapeutic target and encouraged incessant exploring. Gemcitabine was one of the famous RRM1 inhibitors and was approved clinically as first-line drug for anticancer therapy in various cancers. There are other efficient RRM2 inhibitors such as hydroxyurea (HU), already used in the treatment of hematological malignances [26, 27], and Triapine (3AP), evaluated in a number of clinical trials and showed encouraging results in anticancer treatment [28, 29]. However, RRM2 is underexplored and recognized as a viable therapeutic option. With the recent advances in genetic strategies for RRM2 inhibition, some new treatments were reported and highlight the potential of gene therapy. Knockdown of RRM2 through specific small interfering RNA (siRNA) displayed effective antitumor activity in various solid tumors, like head and neck cancer [30], including oral squamous cell carcinoma [31], ovarian cancer [32], gastric adenocarcinoma [33], hepatocellular carcinoma [34], and colorectal cancers [35]. What is more is that therapies targeting or decreasing RRM2 expression through antisense cDNA [36] and RRM2-specific siRNA [37] have displayed a reversal of gemcitabine resistance. Even though the extensive exploration of RRM2 in GBM was rare, Li C. et al. illustrated that RRM2 was overexpressed in human GBM cells and can promote proliferation, migration, and invasion but inhibit apoptosis of GBM cells at experiment level [38]; however, the study only focused on RRM2 simply and the signaling pathway about how RRM2 was involved in was not mentioned. Only Rasmussen et al. demonstrated the RRM2-BRCA1 interaction mechanism and found that BRCA1 protects GBM cells from endogenous replication stress and promotes tumorigenicity [39]. Consistent with previous study, we found RRM2 was strongly high expressed in GBM samples compared to normal brain tissues via comprehensive bioinformation analysis; meanwhile RRM2 was a negative prognostic factor informing worse clinical survival. Importantly, we noted RRM2 may play a role in GBM mainly by effecting cell cycle and nucleotide metabolism; knockdown RRM2 could suppress the proliferation of GBM cells, induce cell arrest in the G1 phase, and promote cell apoptosis [38], supporting present hypothesis. Notably, we identified 20 core genes which were considered to play a central role in GBMs, among which, CDK1, CDC20, CDCA8, CEP55, DLGAP5, KIF20A, KIF4A, NDC80, PBK, TPX2, TTK, VEGFA, and TOP2A had a direct interaction with RRM2; subsequent correlation analysis showed that RRM2 was highly positively correlated with DLGAP5, KIF4A, CDCA8, and TPX2 (R≥0.75, P<0.05). DLGAP5 (Discs Large Homolog Associated Protein 5), also known as HURP or DLG7, is a mitotic spindle protein that promotes the formation of tubulin polymers [40]; it has been documented that DLGAP5 may lead to cancer by allowing cells to progress through the regulation of M phase progression by modulating the function of the spindle apparatus and its organization [41]. KIF4A (Kinesin Family Member 4A) is a microtubule-based motor protein involved in maintaining chromosome integrity during cell mitosis [42, 43]; it was implicated as an potential biomarker in types of cancers [44, 45]. CDCA8 (Cell Division Cycle Associated 8) is one of the components of chromosomal passenger complex (CPC) in mitosis and cell division; in fact, CDCA8 was considered as a putative oncogene for it was overexpressed in tumor tissues but had low or undetectable expression in normal tissues [46, 47]. TPX2 (Microtubule Nucleation Factor) is well known for its critical role in mitotic spindle assembly [48]; a number of studies displayed the efficiency of the strategy of decreasing TPX2 level in anticancer treatment [49]. Moreover, recent works on TPX2 in DNA damage response opened an extended therapeutic window for TPX2-targeted therapies in cancer [50]. Apparently, these genes take part in cell mitosis and cell cycle and are cancer related, even though precise mechanisms of how this PPI network worked are confused; this information adds value to the notion that the RRM2-related network is relevant in carcinogenesis and deserves further exploration in GBM; it may provide a novel avenue worth pursuing when developing effective drugs by combining RRM2 inhibitors (such as 3AP) with other agents. Collecting all, we confirmed that RRM2 is a prognostic factor and a promising therapeutic target for GBM treatment.

## 5. Conclusion

In conclusion, the present study presented a comprehensive bioinformatics analysis of DEGs between glioblastoma and normal tissues and successfully screened several crucial genes and certain associated pathways such as cell cycle and p53 signaling pathway. Our data suggested that pathogenesis and development of glioblastoma are regulated by complex gene network through different biological pathways; RRM2 is a core gene in this network and is associated with the prognosis of glioblastoma; it could be a promising target for efficient suppression of glioblastoma; however, further experiments and more efforts are needed to confirm it and illustrate the specific molecular biological mechanism.

## Figures and Tables

**Table 1:** The GO and KEGG terms enriched by upregulated DEGs.

Category	Term	Count	*P*-Value
GOTERM_BP_FAT	GO:0030198~extracellular matrix organization	14	3.88E-12
GOTERM_BP_FAT	GO:0043062~extracellular structure organization	16	7.24E-12
GOTERM_BP_FAT	GO:0007049~cell cycle	28	2.27E-10
GOTERM_BP_FAT	GO:0000279~M phase	19	3.12E-10
GOTERM_BP_FAT	GO:0007067~mitosis	16	5.21E-10
GOTERM_CC_FAT	GO:0031012~extracellular matrix	21	6.61E-13
GOTERM_CC_FAT	GO:0005578~proteinaceous extracellular matrix	19	1.80E-11
GOTERM_CC_FAT	GO:0005576~extracellular region	42	1.61E-10
GOTERM_CC_FAT	GO:0044421~extracellular region part	28	8.32E-10
GOTERM_CC_FAT	GO:0044420~extracellular matrix part	12	9.61E-10
GOTERM_MF_FAT	GO:0005201~extracellular matrix structural constituent	9	2.58E-07
GOTERM_MF_FAT	GO:0048407~platelet-derived growth factor binding	4	6.59E-05
GOTERM_MF_FAT	GO:0004857~enzyme inhibitor activity	9	9.87E-04
GOTERM_MF_FAT	GO:0050840~extracellular matrix binding	4	0.001069556
GOTERM_MF_FAT	GO:0001871~pattern binding	7	0.001084076
KEGG_PATHWAY	hsa04512: ECM-receptor interaction	10	2.22E-09
KEGG_PATHWAY	hsa04510: Focal adhesion	10	4.37E-06
KEGG_PATHWAY	hsa04610: Complement and coagulation cascades	6	8.15E-05
KEGG_PATHWAY	hsa04110: Cell cycle	5	0.009122958
KEGG_PATHWAY	hsa04115: p53 signaling pathway	4	0.010179414

BP: biological process; CC: cellular component; MF: molecular function.

**Table 2:** The GO and KEGG terms enriched by downregulated DEGs.

Category	Term	Count	*P*-Value
GOTERM_BP_FAT	GO:0007268~synaptic transmission	52	1.62E-31
GOTERM_BP_FAT	GO:0019226~transmission of nerve impulse	54	5.18E-30
GOTERM_BP_FAT	GO:0007267~cell-cell signaling	57	4.96E-21
GOTERM_BP_FAT	GO:0006836~neurotransmitter transport	23	2.20E-18
GOTERM_BP_FAT	GO:0050877~neurological system process	72	3.01E-15
GOTERM_CC_FAT	GO:0045202~synapse	63	1.62E-35
GOTERM_CC_FAT	GO:0044456~synapse part	51	7.00E-32
GOTERM_CC_FAT	GO:0043005~neuron projection	50	4.79E-24
GOTERM_CC_FAT	GO:0008021~synaptic vesicle	25	1.28E-20
GOTERM_CC_FAT	GO:0030054~cell junction	49	1.50E-15
GOTERM_MF_FAT	GO:0022836~gated channel activity	36	6.73E-16
GOTERM_MF_FAT	GO:0005216~ion channel activity	39	3.23E-15
GOTERM_MF_FAT	GO:0015267~channel activity	40	4.98E-15
GOTERM_MF_FAT	GO:0022803~passive transmembrane transporter activity	40	5.43E-15
GOTERM_MF_FAT	GO:0022838~substrate specific channel activity	39	8.74E-15
KEGG_PATHWAY	hsa04080: Neuroactive ligand-receptor interaction	25	3.52E-10
KEGG_PATHWAY	hsa04020: Calcium signaling pathway	21	4.34E-10
KEGG_PATHWAY	hsa04720: Long-term potentiation	9	8.19E-05
KEGG_PATHWAY	hsa04012: ErbB signaling pathway	8	0.00232
KEGG_PATHWAY	hsa04010: MAPK signaling pathway	13	0.010508

BP: biological process; CC: cellular component; MF: molecular function.

**Table 3:** The statistical results of the connectivity of the network.

Gene	MCC	DMNC	MNC	Degree
TOP2A	9.22E+13	0.87325	40	56
CDK1	9.22E+13	0.68829	47	47
VEGFA	5763	0.18902	40	43
SYT1	4.37E+07	0.37425	40	42
CDC20	9.22E+13	1.01161	34	37
KIF4A	9.22E+13	0.98669	34	36
CALB1	4586	0.27262	33	35
BDNF	7597	0.33146	32	35
SYP	3760934	0.39583	33	35
DLGAP5	9.22E+13	1.1534	33	35
KIF20A	9.22E+13	1.05547	35	35
TTK	9.22E+13	1.10629	34	35
NDC80	9.22E+13	1.13243	33	35
GNG3	7267294	0.41708	32	34
CEP55	9.22E+13	1.07639	34	34
SYN1	3799485	0.41397	31	34
PBK	9.22E+13	1.09881	34	34
CDCA8	9.22E+13	1.1038	34	34
TPX2	9.22E+13	1.11408	33	34
RRM2	9.22E+13	1.03281	33	34

**Table 4:** The GO and KEGG terms enriched by 20 hub genes.

Category	Term	count	*P*-Value	Genes
GOTERM_BP_FAT	GO:0000279~M phase	9	6.73E-09	CDK1, CDCA8, DLGAP5, TPX2, TTK, CDC20, NDC80, CEP55, PBK
GOTERM_BP_FAT	GO:0000280~nuclear division	8	1.17E-08	CDK1, CDCA8, DLGAP5, TPX2, CDC20, NDC80, CEP55, PBK
GOTERM_BP_FAT	GO:0007067~mitosis	8	1.17E-08	CDK1, CDCA8, DLGAP5, TPX2, CDC20, NDC80, CEP55, PBK
GOTERM_BP_FAT	GO:0000087~M phase of mitotic cell cycle	8	1.32E-08	CDK1, CDCA8, DLGAP5, TPX2, CDC20, NDC80, CEP55, PBK
GOTERM_BP_FAT	GO:0048285~organelle fission	8	1.54E-08	CDK1, CDCA8, DLGAP5, TPX2, CDC20, NDC80, CEP55, PBK
GOTERM_CC_FAT	GO:0015630~microtubule cytoskeleton	10	1.60E-08	CDK1, CDCA8, KIF4A, DLGAP5, TPX2, TTK, CDC20, CEP55, TOP2A, KIF20A
GOTERM_BP_FAT	GO:0000278~mitotic cell cycle	9	1.69E-08	CDK1, CDCA8, DLGAP5, TPX2, TTK, CDC20, NDC80, CEP55, PBK
GOTERM_CC_FAT	GO:0005819~spindle	7	3.46E-08	CDK1, CDCA8, KIF4A, DLGAP5, TPX2, TTK, CDC20
GOTERM_BP_FAT	GO:0022403~cell cycle phase	9	4.04E-08	CDK1, CDCA8, DLGAP5, TPX2, TTK, CDC20, NDC80, CEP55, PBK
GOTERM_BP_FAT	GO:0022402~cell cycle process	9	4.43E-07	CDK1, CDCA8, DLGAP5, TPX2, TTK, CDC20, NDC80, CEP55, PBK
KEGG_PATHWAY	hsa04110: Cell cycle	3	0.008429	CDK1, TTK, CDC20
KEGG_PATHWAY	hsa04115: p53 signaling pathway	2	0.077638	CDK1, RRM2

BP: biological process; CC: cellular component; MF: molecular function.

## Data Availability

The data used for analysis in this study are available from the Cancer Genome Atlas and the Gene Expression Omnibus database freely.

## Supplementary Materials

Supplementary Materials Supplementary Figure 1: correlation between the expression of RRM2 and other hub genes. Supplementary Table 1: correlation between the expression of RRM2 and other hub genes.
