# Supplementary material for: Identification of Potential Biomarkers in Glioblastoma through Bioinformatic Analysis and Evaluating Their Prognostic Value
Source: Biomed Res Int. 2019 Apr 15;2019:6581576. doi: 10.1155/2019/6581576 (PMC6500689; doi:10.1155/2019/6581576)
Supplement: Supplementary Materials — Supplementary Figure 1: correlation between the expression of RRM2 and other hub genes. Supplementary Table 1: correlation between the expression of RRM2 and other hub genes. [file 6581576.f1.pdf]

Supplementary Figure 1. Correlation between the expression of RRM2 and other hub genes.

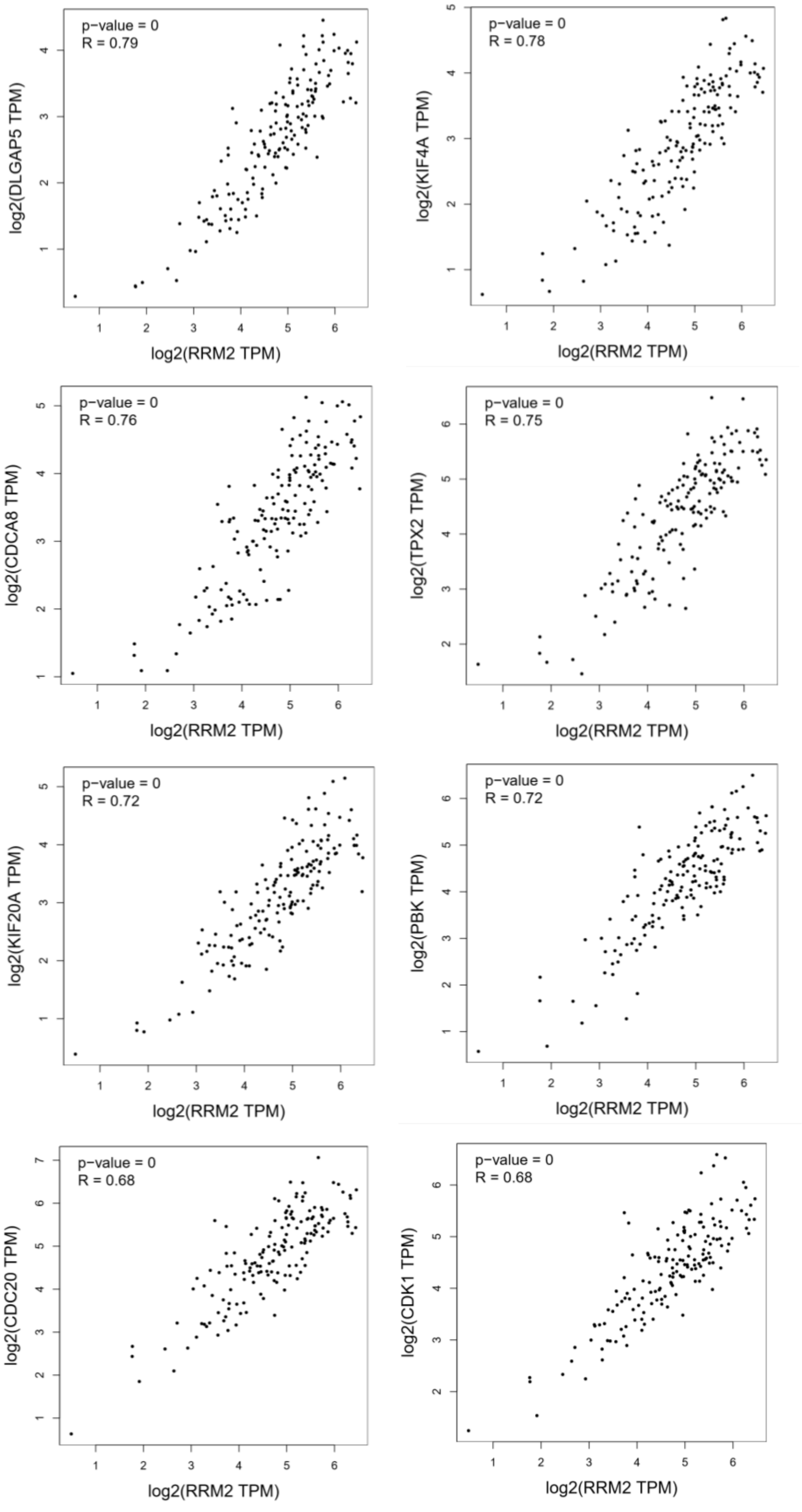

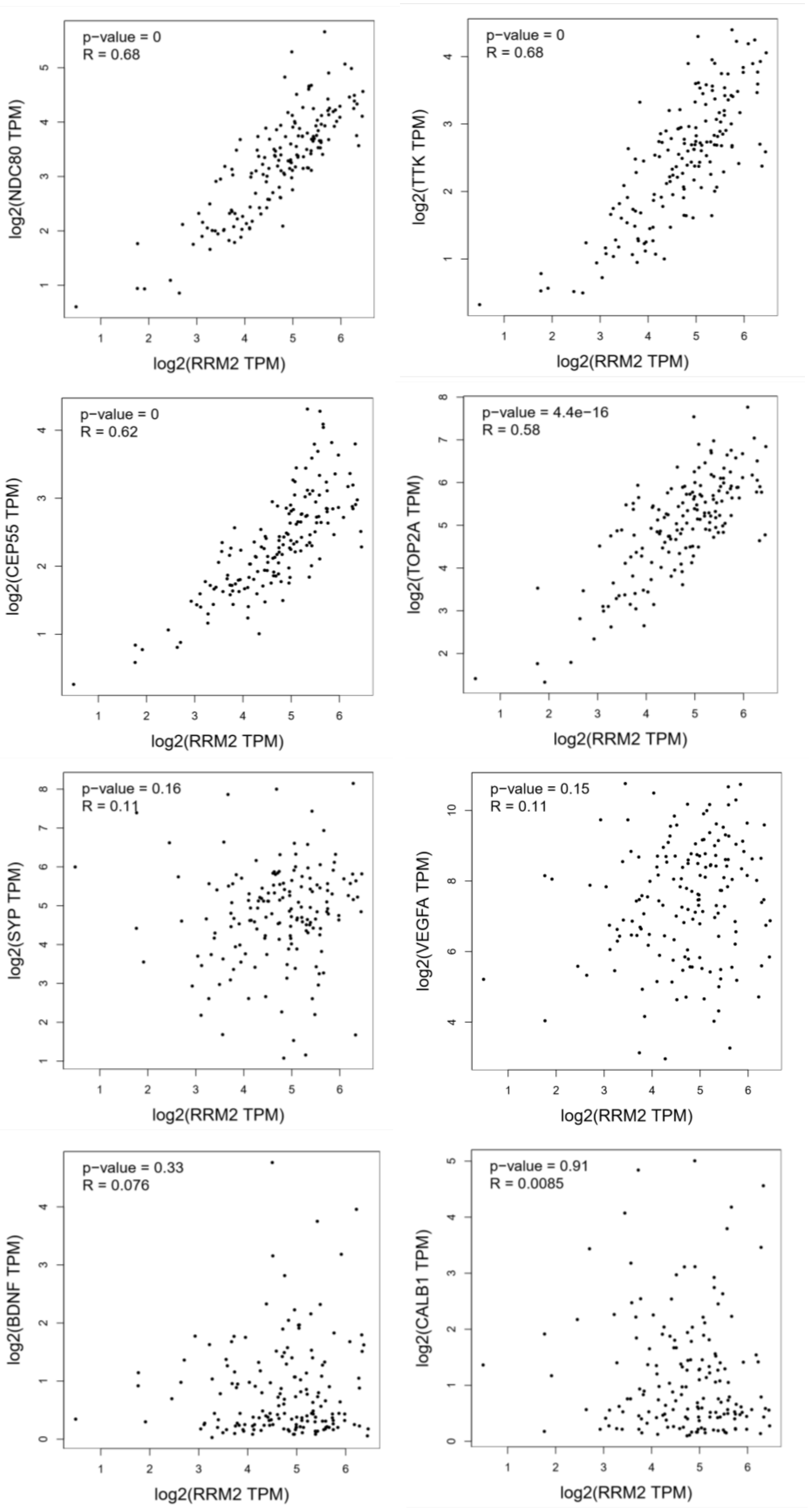
p−value = 0
R = 0.68
log2(NDC80 TPM)
log2(RRM2 TPM)
p−value = 0
R = 0.68
log2(TTK TPM)
log2(RRM2 TPM)
p−value = 0
R = 0.62
log2(CEP55 TPM)
log2(RRM2 TPM)
p−value = 4.4e−16
R = 0.58
log2(TOP2A TPM)
log2(RRM2 TPM)
p−value = 0.16
R = 0.11
log2(SYP TPM)
log2(RRM2 TPM)
p−value = 0.15
R = 0.11
log2(VEGFA TPM)
log2(RRM2 TPM)
p−value = 0.33
R = 0.076
log2(BDNF TPM)
log2(RRM2 TPM)
p−value = 0.91
R = 0.0085
log2(CALB1 TPM)
log2(RRM2 TPM)

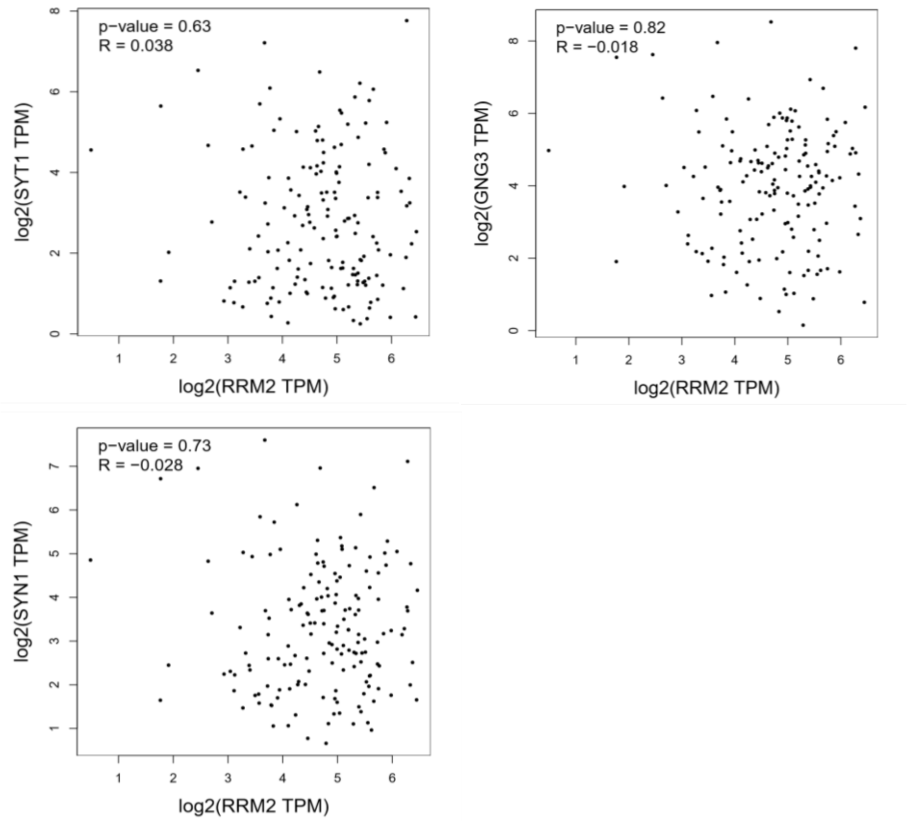

Supplementary Table.1 Correlation between the expression of RRM2 and other hub genes.

gene	R	P
DLGAP5	0.79	0
KIF4A	0.78	0
CDCA8	0.76	0
TPX2	0.75	0
KIF20A	0.72	0
PBK	0.72	0
CDC20	0.68	0
CDK1	0.68	0
NDC80	0.68	0
TTK	0.68	0
CEP55	0.62	0
TOP2A	0.58	4.40E-16
SYP	0.11	0.16
VEGFA	0.11	0.15
BDNF	0.076	0.33
CALB1	0.0085	0.91
SYT1	0.0038	0.63
GNG3	-0.018	0.82
SYN1	-0.028	0.73
